# Exploring health literacy in relation to noncommunicable diseases in Samoa: a qualitative study

**DOI:** 10.1186/s12889-019-7474-x

**Published:** 2019-08-22

**Authors:** Caroline Bollars, Kristine Sørensen, Nanne de Vries, Ree Meertens

**Affiliations:** 1 Department of Health Promotion, School of Nutrition and Translational Research in Metabolism (NUTRIM), and Care and Public Health Research Institute (CAPHRI), Faculty of Health, Medicine and Life Sciences, Maastricht University, PO Box 616, 6200 Maastricht, The Netherlands; 2 Global Health Literacy Academy, Risskov, Denmark

**Keywords:** Health literacy, Public health, Health promotion, Focus groups, Medication adherence, Noncommunicable diseases, Risk factors, Obesity, Diabetes mellitus, Qualitative research, Primary health care, Samoa

## Abstract

**Background:**

Samoa is suffering from alarming rates of noncommunicable diseases (NCDs). To address this epidemic, tackling health literacy is important. A qualitative study was conducted with the aim to explore health literacy in Samoa in relation to NCDs.

**Methods:**

Six focus groups were conducted, with a total sample size of 73 participants aged over 18 years. The semi-structured interview guide was based on the conceptual model of the European Health Literacy project (HLS-EU). Data was translated, transcribed, coded, and categorized as part of the qualitative analysis.

**Results:**

The analysis resulted in one overarching category and seven sub-categories based on 19 themes. It revealed that health literacy in Samoa is strongly influenced by the culture. Personal responsibility is lacking. The family circle is central to health in a community where support is provided through the church and local groupings. Basic knowledge of NCDs was present in the population, but a deeper understanding of chronic disease implications was lacking. Difficulties with regards to medication adherence for chronic diseases arose as a topic, and traditional healers are still strongly embedded in the local society. Finally, the health system’s performance, especially primary care services at the local level, is suffering from the high burden of NCDs and has been challenged to respond to the needs of the community it serves.

**Conclusion:**

The findings of this study show how health literacy in Samoa is influenced by culture and suggest employing participatory, culture-sensitive, public health interventions which address the family as a whole, building on health literacy to address major public health problems like NCDs and remove barriers in the health system.

## Background

Samoa is suffering from alarming rates of noncommunicable diseases (NCDs). The Island Nations and Territories in the South Pacific, including Samoa, have an epidemiological profile showing high estimates of overweight and obesity as well as high blood pressure and blood sugar. Diabetes mellitus type II and its complications, cardiovascular disease and hypertension feature prominently in the leading causes of both morbidity and premature mortality [1]. The latest evidence confirms that chronic diseases such as NCDs are responsible for 70% of deaths globally. Noncommunicable diseases are the result of a combination of genetic, physiological, environmental and behavioural factors. They disproportionately affect people in low- and middle-income countries where more than three-quarters of global “premature” NCD deaths – 31 million – occur [2]. Risk factors of NCDs as well as its co-morbidities target predominantly the adult population resulting in life changing circumstances for its social environment, the family and community [3].

### Exploring health literacy in relation to NCDs

Recent research in public health has illustrated the importance of health literacy with regards to addressing disease burdens such as NCDs. Health literacy is linked to literacy and entails the motivation, knowledge and competence to access, understand, appraise and apply health information to make judgements and take decisions concerning healthcare, disease prevention and health promotion in everyday life during the life course [4]. Health literacy is a relational concept which focuses on the skills of individuals as well as on the complexity of services and systems and how they interact [5]. Hence, health literacy is about having the knowledge, confidence and skills to seek out and process information from a variety of sources to improve and protect health [6]. However, in the Pacific Island Countries and Territories, little is known about health literacy.

Generalization from health literacy interventions in high-income countries may be hindered by cultural, social and situational factors specific for small island developing states. Therefore, a qualitative study was conducted to explore health literacy in Samoa in relation to NCDs to provide insights on how health is perceived, with specific attention paid to the high burden of NCDs.

### The South Pacific Island of Samoa

Samoa is an independent, Polynesian Pacific island country struggling to address the NCD epidemic at the health system, community and individual levels. Samoa’s small population size, remoteness and vulnerability to natural disasters constrain economic growth, and as a result its small economy is highly dependent on tourism, remittances and external development assistance [7]. Regarding general literacy, although enrolment in primary education in Samoa is high, the Samoa Pacific Islands Literacy and Numeracy Assessment results give cause for concern, as only 8% is performing satisfactory at the Grade 6 Literacy level [8].

NCDs account for 75% of the total disease burdens in 2016 and more than half of all premature deaths in the country. NCDs are also the major driver of overseas medical treatment with overseas treatment accounting for 10–15% of the Total Health Expenditure. The share of total health expenditure from private expenditure is low, an estimated 15% of total health expenditure comes from the private sector in Samoa, compared to 54.6% in other lower-middle-income countries. The health financing challenge is that expenditure is still largely focused on inpatient curative care more than primary and secondary preventive care. The current service delivery system in Samoa is heavily hospital-centric characterized with weak primary health care structure and overcrowding in the main referral hospital. Samoa suffers from an effective care model for NCDs. Gaps include low screening rate, weak follow-up and referral, as well as lack of a patient tracking system. As a result, most of the NCDs patients in Samoa have not been detected, diagnosed and put under regular disease treatments. Without effective disease management, the disease will further progress to comorbidity such as stroke, cardiovascular diseases and kidney failure. The country faces a high burden of pre-mature death and considerable increase of kidney dialysis cases [9]. The Government’s Health Sector Strategy recognizes NCDs as an increasing problem and has taken important actions to address the challenge. Revitalizing primary care and strengthening community engagement in the control of NCDs is one of those actions. The work of this research aims to support the strengthening of community engagement within the NCD control and hopes to contribute to this work. [10]. It has become clear that managing chronic diseases such as NCDs demands a shift in the responsibility from the patients as passive receivers to individuals as active actors in their own health [11]. In order to be able to cope with this shift of responsibility, it is crucial that public health interventions are built up with evidence-supported guidance on how to increase health literacy and to effectively address the NCD epidemic. Therefore, the aim of this study was to explore the health literacy of Samoans and guide public health experts and policy makers to understand key components of health literacy in relation to NCDs and the role it plays for the development of a timely as well as appropriate response from the perspectives of health services as well as patients and citizens.

## Methods

### Study design and participants

The study utilized focus groups as a qualitative research approach to explore health literacy in Samoa.

A non-probability purposive cluster sampling method was chosen. The population of Samoa was divided into clusters following the cluster approach conducted for the census survey by the Samoa Bureau of Statistics. Samoa is divided into four statistical regions (Apia urban area (1), North West Upolu (2), Rest of Upolu (3) and Savaii (4)). Within these regions convenience sample clusters of villages were chosen. The study sites selected covered a total of seven villages with a representation of the four different regions in Samoa. Purposive sampling was used to select participants for the study. Inclusion criteria were: rural background (living in a village) as well as being aged above 18 years. The participants were approached by the researchers with the approval of the Ministry of Women Community and Social Development and the Ministry of Health. The village committee served as a formal community gatekeeper [12]. The villages were selected according to their geographical spread from the main village of Apia to ensure rural representation and distribution across the country. The research team contacted the village committees, which consented to the recruitment of participants and invited village members on a voluntary basis. Then the research teams were given a list of village members who were willing to participate. The facilitator was recruited through the WHO Office in Samoa as an expert for conducting focus group, with a key expertise in qualitative research. The facilitator met the research team in advance and discussed sampling as well as the villages. The participants were not related or known to the facilitator. The venue of the focus groups was discussed in detail with the facilitator and research team. Several options were carefully assessed such as venue in Ministry of Health, venue within the village (church or women committee’s house and the WHO office in Apia, Samoa. The option of the WHO Office was considered as most neutral. It should be noted that the WHO Office in Samoa is low profile, located in a simple office layout with limited resources available.

### Interview guide

The methodological orientation and theory utilized to underpin this study was derived from the conceptual model of the European Health Literacy project (HLS-EU) [13]. The research team (CB, KS and RM) developed a semi-structured interview guide for the conduct of the focus groups based on the conceptual model of the HLS-EU. The conceptual model focuses on how people access, understand, appraise and apply information to form decisions in everyday life concerning healthcare, disease prevention and health promotion. It also shows how health literacy is influenced by personal, situational and societal determinants. It indicates how health literacy impacts health service use and costs; health behaviour and health status; social participation and empowerment; as well as equity and sustainability [13]. The interview guide also contained queries related to local Samoan customs and conditions. It is listed in Table 1.

**Table 1:** Interview guide based on conceptual model of HLS-EU

Theme of HLS-EU	Questions
Health care	• What have you heard about diabetes? • What do you know about diabetes? • Why is it important? • How easy or difficult is it for you to find information on diabetes? • What have you heard about high blood pressure (hypertension)? What do you know about high blood pressure? • How easy or difficult is it for you to find information about high blood pressure? • How difficult is it to access the health facility when you or family member is suffering from diabetes or hypertension? • How can diabetes be treated? How can high blood pressure be treated? • How well do you understand the information you receive concerning diabetes and high blood pressure? • How can you judge if this is relevant for you, or for members of your family or your friends?
Disease prevention	• What can be done to prevent chronic conditions such as e.g. diabetes and high blood pressure? • What makes it difficult to prevent these chronic conditions? • What can help overcome the barriers? • What can help motivate you to prevent diabetes and high blood pressure?
Health promotion	• Where do you usually learn about health? • Who impacts how think about health? (e.g. your spouse, your mother, the doctor, the news …) • How would more information help you to follow the guidelines on keeping healthy? • How well do you understand the health advices that are provided? • How do you judge if the health information is relevant to you? • Whom do you trust to provide the best health advice? • How do you know what to do to improve your health or your medical conditions?
Personal factors	• What personal factors influence diabetes? • What personal factors influence high blood pressure? • How do personal factors influence the development of non-communicable diseases?
Situational factors	• How does the local community impact the development of diabetes? High blood pressure? • How does the local community prevent diabetes? High blood pressure?
Societal factors	• How does the society in Samoa influence the development of diabetes? High blood pressure? • How does the society prevent diabetes? High blood pressure?
Health costs	• What are the cost implications of having diabetes? High blood pressure?
Lifestyle/health behaviour	• What are the impacts of diabetes on your lifestyle? On your health behavior? • What are the impacts of high blood pressure on your lifestyle? On your health behavior?
Participation	• How does diabetes impact the participation in everyday life activities? and social activities? • How does high blood pressure impact the participation in everyday life? and social activities?
Socio-economic factors/sustainability	• How does diabetes impact the financial sustainability? • How does high blood pressure impact the financial sustainability? • How does diabetes impact the ability to work? • How does high blood pressure impact to work?

### Focus group interviews

The focus groups were facilitated by the trained facilitator and were conducted in the Samoan language. The principal investigator (CB) participated in all focus groups as an observer. After each focus group, the facilitator and the researcher made notes and discussed the preliminary observations. The focus groups took place from August 2015 to November 2015 in the meeting room of the local WHO Country Office for Samoa except for two of them, located on the island of Savaii, where the church served as the meeting site. Each focus group lasted approximately 2.5 h.

The guidelines for retrieving informed consent were followed, and consent was obtained from all participants in the focus groups. A short discussion took place before each focus group interview to explain the process and provide clarification. The Ministry of Health provided ethical approval through the Health and Research Committee. Two representatives of the Ministry of Health were present in the focus groups as non-participants as the research will support a future intervention to address NCDs [14].

### Transcription and translation

The interviews were audio-recorded and transcribed as preparation for the in-depth data analysis. A native Samoan speaker translated the transcripts into English, and another bilingual speaker did the back-translation. When there were differences, the primary researcher (CB) finetuned them with the Samoan native speaker who also participated in the focus groups.

### Data analysis and data saturation

The data was manually coded by two independent coders (CB and KS). The transcripts (quotations, sentences or words) were labelled with codes to help catalogue key concepts while preserving the context in which these concepts occurred. Hereafter, the codes were inductively clustered into themes and categories that were compared and discussed to yield an overview of the trends in the views regarding health literacy in Samoa put forward by the focus groups. In the clustering process, it was realized that the data was not saturated enough to deductively cover all the themes of the conceptual model which formed the basis of the interview guide was. Instead, an inductive analytical strategy was chosen to yield the best possible overview of the emerging trends based on saturated themes.

An audit trail of field notes, logbook and analytical notes was kept through the research process to facilitate transparency. The data was kept by the principal researcher and shared only with the co-authors of this manuscript, data security was ensured. Confidentially was maintained through assigning unique ID codes to the participants in the data set and only the principal researcher was familiar with the unique coding ID.

## Results

The study is based on six focus groups with a total of 73 participants; the focus group size varied from 11 to 14 participants and the mean age was 48 years. Table 2 details the demographic characteristics of the focus groups participants. In terms of household size, the value was 6–10 members above 18 years of age per household. Regarding income, the majority of the participants reported to have less than 200 Western Samoa Tala (WST) per week, equivalent to 77 USD per week or 330 USD per month.

**Table 2:** Demographics characteristics per focus group (FG)

Demographic	FG1	FG2	FG3	FG4	FG5	FG 6	FG total
Total participants	11	12	11	14	13	12	73
Gender	11 F	10F; 2 M	8F; 3 M	12F; 2 M	9F; 4 M	F8; 4 M	58F; 15 M
Mean age	50	62	42	44	40	52	48
Median household size	6–10	6–10	11–20	6–10	6–10	11–20	6–10
Median income, Western Samoa Tala (WST)	< 200 WST	< 200 WST	< 200 WST	< 200 WST	< 200 WST	< 200 WST	< 200 WST

**Fig. 1:**
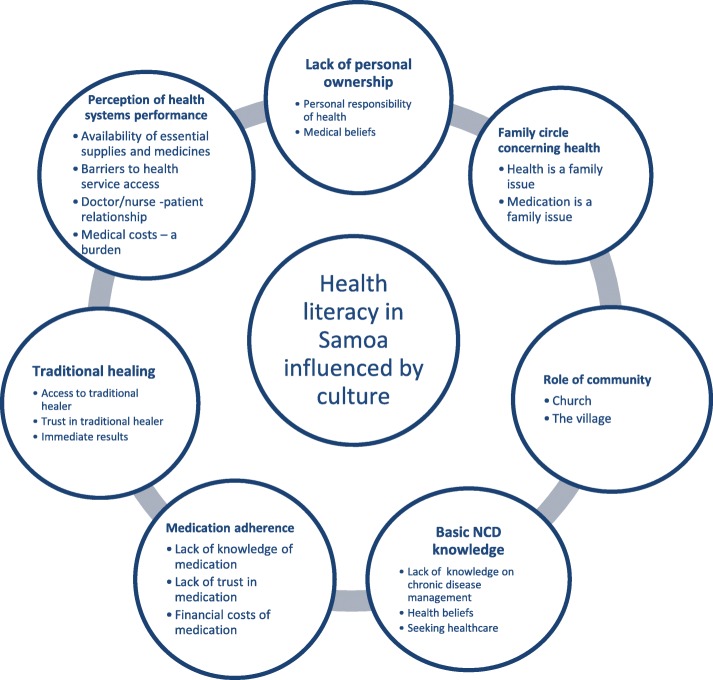
Seven categories with 19 themes that were revealed by the focus group data (grouped around the central trend)

The results of the inductive analysis revealed 40 themes. After removing duplicates, a total of 19 themes were clustered into one main category and seven emergent sub-categories Fig. 1.

Overall, the essence of the results showed that health literacy in Samoa is strongly influenced by the culture. The personal ownership of health is lacking as the family circle is influential as well as the local community that provides support through the church and local groupings. Furthermore, basic knowledge on NCDs is present in the population, but a deeper understanding of chronic disease implications is needed. Difficulties regarding medication adherence for chronic diseases arose as an important topic, and traditional healers were noted to be strongly embedded in the local society. Finally, the health system’s performance, especially primary care services at the local level, has suffered from the high burden of NCDs and has been challenged to respond to the needs of the community.

Thick descriptions of the categories with quotes from the focus groups (FG) are provided below.

### Lack of personal ownership

The lack of personal ownership of health was a category that appeared strongly with two emerging themes: personal responsibility of health and medical beliefs. Samoans tend not to judge from personal beliefs but always live and act according to the community rules. During the focus groups, a participant stated: “*Health should be a personal responsibility; however, we as Samoans do not want to be responsible for our health*” *(FG2).*

Participants referred to it as being spoiled or lazy.

*“Samoans are like babies; they want to be spoon-fed all the time” (FG3).*

*“We want to wait around for others to come and hand us everything we need” (FG1).*

*“People are lazy” (FG4).*

*“But they need to know that their health is their own personal responsibility” (FG4).*

The theme of medical beliefs and opinions was widely discussed in all focus groups.

Participants stated: “my belief of treating myself or family is coming from what we believe we should do based on the community values” (FG6).

*“My opinion does not count for my own health, I do what is best as I have seen others doing” (FG3).*

*“What is happening with all these diseases coming from abroad is not my problem, it is given to us by all these foods. It is my belief that I cannot do anything about it” (FG1).*

### Family circle concerning health

The second category detailed themes describing the importance of the family circle with regards to health. It is based on two themes: health is a family issue as well is medication. Firstly, the study showed that health is considered a confidential and family-related issue in Samoa, and it is perceived as “*bad manners*” to speak about your own health issues outside the family.

*“In Samoan culture, we do not speak about private issues such as your health problem or your disease” (FG3). “Health is only discussed within the family circle” (FG6).*

The study pointed out that family factors are very important in relation to one’s health. Personal decision-making around health is perceived as a challenge as health decisions are considered family decisions.

*“Samoans cannot do it on their own” (FG2). The family approach including the support to achieve the desired outcomes is essential in the Samoan culture.*

*The Samoan people do not seek healthcare easily and if we seek care, we always come as a family, not as an individual” (FG1).*

*“A whole family approach needs to be used as we always decide together” (FG3).*

*“Everyone needs to work together to achieve desired results” (FG5).*

*“High blood pressure can be treated by making sure your family provides the support” (FG6).*

Secondly, the study revealed that taking medication is also a family issue. The family component, is one of the cues for action described as an enabling factor to manage medication.

*“Taking medication is difficult for Samoan people; people do not know the names of their medication nor what type of medication they are taking and for what reason” (FG1).*

*“A younger member of the family should be in charge of medication, I cannot do it on my own” (FG5).*

*“It should be the whole family to remind the diabetic family member to take their pills when it is time to do so” (FG2).*

### Role of community

The category regarding the ‘Role of community’ included themes related to church and village groupings. Health-related decisions in Samoa are strongly influenced by peers and/or community leaders. As a general theme emerging, the church is considered an important social setting within the community and plays a significant role in people’s health. *“As church members, we dance during praise and worship so that has also a positive effect on my health” (FG5).*

In addition, the village as a formal setting was perceived as a factor for supporting a healthier life as the Samoan society is built around community participation. However, the potential for health promotion was rarely used, only when the doctor visited the village.

*“It should be discussed in the village community” (FG2).*

*“We do not talk about health issues at the community level, only when the doctor visits the village, but that rarely happens” (FG6).*

The fa’atofalaiga or the traditional Samoan way of informing and agreeing on activities within the village setting was mentioned. In line with keeping up with Samoan culture, consultations are conducted with community representatives: village chiefs (Matai), church leaders and women’s committee leaders. Mass drugs administration on lymphatic filariasis through the church was given as an example: “*for this other disease with the limbs, our pastors have preached and told us to take the pills as a whole family. After service, the nurse was waiting and handing us the pills. We felt safe as our pastor guided us. (FG3)* “.

### Basic noncommunicable diseases (NCDs) knowledge

The study revealed that participants had a basic knowledge of NCDs and were aware that Samoa has been active in recent years promoting health information campaigns set out by the Ministry of Health on conditions such as overweight and obesity, hypertension and diabetes. However, it was highlighted that the health communication focused on NCD risk factors and not directly on disease management. Although health information has been provided to the general public in recent years on the importance of physical activity, non-smoking and a healthy diet, it seems there is still a lack of connection with the conditions themselves.

*“I know about basic measures” (FG2).*

*“I am aware of diabetes and high blood pressure” (FG3).*

*“I know that when somebody with diabetes has an attack you should give a lolly” (FG4).*

A common theme appearing in the study concerned the health beliefs of Samoans. Various beliefs regarding NCDs and their importance were prevailing.

*“I believe this diabetes cannot be treated” (FG4).*

*“Diabetes will lead to a person losing a limb at some point, nothing to do about it” (FG5).*

*“Family members that have it can give it to you” (FG6).*

Seeking healthcare for a condition in which one does not feel any physical or direct discomfort is not a common practice in Samoa. People seek health care when their condition is urgent and not for invisible symptoms.

*“I am putting off going to see the doctor” (FG1).*

*“I prefer to forget about it” (FG3).*

*“I only go to the hospital when it becomes an emergency case” (FG6).*

*“It is up to the person to go and see a doctor if they suspect that their health is bad” (FG2).*

### Medication adherence

Medication adherence is the fifth category, which revealed that the attitude towards it was poor. There is a crucial lack of knowledge regarding the prescribed medication. Prescribed medication is dispensed to the patient in bulk at the primary health care centre. Neither the prescription nor the dosage is detailed. The patient is directly dependent on the health professional as the sole source of information regarding the prescribed medication.

*“People need to know the names and kinds of pills they are taking” (FG4).*

*“It is difficult to check the pills you have to take, because you forget” (FG6).*

*“Too complicated all these pills” (FG3)*

*“We do not understand what we have to do” (FG5).*

The study indicated, that in Samoa, medication is considered needed only when the individual is suffering or displaying visible signs of malfunction.

*“If I feel better, I will stop to take my medication as I believe this is better for me” (FG1).*

Another general theme is related to the lack of trust. Samoans do not trust in the performance of the health system (see below) nor do they trust the medication provided.

*“I do not really trust the medication” (FG2).*

*“You drink the pills yet they never seem to get you better” (FG6).*

*“I believe it will introduce poison in my body which will affect me so I do not take the medication” (FG2).*

*“I do not like taking pills” (FG2). “I just will not take them” (FG2).*

*“I do not want to go to the hospital anymore because every time I go, I get pills” (FG5).*

Another barrier for medication adherence was the burden of financial costs related to medication.

*“I can’t afford the pills” (FG5).*

*“Cost too much money these medicines” (FG1).*

### Traditional healing

The sixth category that appeared was called traditional healing. The themes identified were related to the easy access, trust and immediate results the community perceived with the traditional healers. The availability of traditional healers in the village and the trust they experience (within the community they are considered wise seniors) was a reason why traditional healers were still considered a good resource for care.

*“Traditional healers were mainly used in the past for any ailments” (FG2).*

*“People are more aware that they need to go and see a doctor for things that are beyond a traditional healer’s knowledge” (FG4).*

Additionally, chronic diseases like diabetes and cardiovascular diseases are considered by some as Samoan ailments, which can be cured with a massage by traditional healers using herbal leaves. Indications of such beliefs, that immediate results can be obtained in the treatment of a chronic disease, were quite common.

*“There is still trust in traditional healers” (FG6).*

*“If you go to a traditional healer, you can get immediate results” (FG3).*

*“Go to the fofo (traditional healer) to get massage and herbal drink to keep you strong” (FG5).*

*“The traditional healers are right in the middle of our village and can always give good advice” (FG1).*

### Perception of health system’s performance

The themes clustered under this category showcased the individual’s direct experiences with healthcare and perceptions as a result of healthcare interaction. The themes focused on the availability of essential supplies and medicines, barriers to healthcare service access, medical costs, and the doctor/nurse-to-patient relationship. It became evident that the perception of the health system’s performance is an important factor with regard to chronic disease management in Samoa. Many participants perceived the health system as being too expensive, having too few resources (e.g. supplies and medicines), and limited healthcare staff who were sometimes unfriendly.

*“The local hospital has limited staff and no resources, why should I go there” (FG6)?*

*“When I visit, I have to wait many hours and often the nurse could not give me my medicine because they did not have the supplies in the hospital” (FG4).*

*“When I go to the hospital, they have always run out of strips to do the blood test for my aunt’s diabetes” (FG4).*

*“I have to wait for too long to be seen by a doctor” (FG2).*

*“I waste so much time just waiting” (FG3).*

The limited human resources of primary healthcare staff were perceived to be a barrier for help.

*“The local hospital cannot do anything” (FG1).*

*“I cannot ask the doctor any questions, always too busy” (FG5).*

*“In the hospital, the nurse can give the advice but is always too busy” (FG3).*

Finally, a general theme described was the doctor/nurse - patient relationship. The attitude towards the patients was perceived as negative and rude.

*“The nurses tell you off in front of everyone” (FG5).*

*“Some nurses are very rude” (FG1).*

*“Staff always serve people they know or their family members first, rather than first come, first serve” (FG2).*

## Discussion

The aim of this study was to explore health literacy in Samoa in relation to NCDs. Limited research is available regarding health literacy in developing countries. The global burden of disease study from 2015 found that the epidemiological disease burden from NCDs is more prevalent in low economic settings [15]. In Samoa, NCDs are high among the major causes of premature mortality, but little is known about the health literacy of its population.

The study helped to describe how health literacy is influenced by culture; particularly in Samoa, the family is a key pillar in terms of the management of illness. The patients depend on their family members as carers to help, teach and remind them about managing their own condition. Especially with chronic conditions such as NCDs, which demand a long-term care solution, this finding can support patients in addressing the struggles faced by the health care services to deal with the NCD epidemic. The Samoan society functions as a family, the “Aiga”. Any health service facility operating in Samoa will have not only the patient waiting but their family members waiting together with them. The findings show the importance of culture and underline the significance of the recommendation of the researchers from the Ophelia studies to investigate specific health literacy needs in the local community [16]. As NCDs demand long term care solutions, it entails the individual to commit and comprehend the specific care required for them to proactively engage in the control of their own health [17].

The present study revealed the use of both traditional healers and official health services. Research on the utilization of certified health care services in low income countries pointed out the inter-relationship of people’s perception with regards to effective treatment and their decision in terms of accepting or rejection the services available to them [18]. Once again, the results of this study confirmed the importance of culture in health. People participate in primary health care to the extent that they experience the service as offering reliable solutions to what they perceive as health problems, and this is exactly in line with the findings of this study. The decision of whether to use the services at the beginning of an illness, later, or even at all is calculated by weighing the anticipated benefits (relative to other alternatives) against the expected expenditure of time and money, and the personal abuse or friendliness of health workers that these efforts may entail. Understanding how people estimate factors on both sides of the equation relating costs to benefits is crucial to understanding the demand for primary health care services and, as a corollary, the strengths and failings of the programmes [19]. A Lancet Commission in 2014 published a paper on the importance of the role of culture in health, and it has shown how inseparable health is from the cultural perception of wellbeing [20].

Lack of human resources and medical supplies at the health facility and lack of financial resources were recurring major issues and recorded as a challenge in terms of health system performance. It is questionable what impact an intervention to address health literacy will have if financial hardship is one of the key barriers to accessing and seeking healthcare. It is also of importance to address broader societal determinants of health such as financial hardship and to ensure these basic values and rights are fulfilled as detailed in the recent call for Universal Health Coverage put forward by the Tokyo Declaration [21]. Samoa graduated from Least Developed Country to Developing Country in January 2014. This graduation shows an improvement at the macro-economic level, with anticipated annual growth of real GPD. In 2014, the United Nations estimated that Samoa would continue to further increase its human capital as measured by the Human Asset Index, experiencing improvements in child mortality, literacy and secondary school enrolment [22].

The study showed that a basic knowledge of NCD prevention is present, but it seems disconnected from a deeper understanding of the chronic impact of the diseases and their management. In terms of disease knowledge and awareness, chronic diseases such as NCDs might be perceived like any other disease, and therefore the implications of why people should go and see a doctor if they do not feel ill is misunderstood. The WHO Commission on Social Determinants of Health stated that vulnerable groups are likely to suffer worse health outcomes, and the recent literature is now associating this with lower levels of health literacy [23]. Public health interventions focussing on the early detection and management of NCDs should embark on a broader understanding of how health literacy is influenced by culture.

In the findings of this study, it became apparent that health care professionals are perceived as rude, and some crucial gaps were highlighted in the nurse/doctor-to-patient relationship. In attempts to understand why people in developing countries under-use health services, the uncongenial atmosphere in clinics and unfriendly service by clinicians have been documented in the ethnographic literature since the 1950s and 1960s [24]. The same barriers were identified in this study. In addition to the better-known issue that patients and doctors, the latter operating from a medical perspective, might not view illness and its treatment similarly, anthropologists have noted that the treatment clients receive is often abusive [25]. In Samoa, more research should be conducted into health care utilization and traditional beliefs and practices.

In general, the results pointed out that in Samoa, people have a holistic view of health: a person is healthy when he or she is in tune with his/her environment and community. One’s ability to cope with the environment/community determines the state of wellness/health, and one’s ability to cope is in turn strongly dependent on one’s knowledge of culture. Being part of the traditional community and closely identifying with the culture of where one belongs is part of surviving skills to deal with unexpected events in life such as diseases. This knowledge allows a person to understand their place in the bigger scheme of things [26]. Therefore, effective interventions to promote the ability to cope with health challenges are clearly determined by the knowledge of the culture in Samoa.

Applying the public health approach of health literacy suggests building a bottom-up, participatory, public health intervention that consists of three layers of health literacy: knowledge and skills regarding health information and health systems; social skills to enable interaction within the health system environment; and lastly, the critical ability to understand and apply the information received as relevant to one’s own health [27].

The findings of this study detail novel Samoan results on how health literacy is influenced by culture and suggest that experts and policy makers should employ participatory, culture-sensitive, public health interventions to address major public health problems like NCDs by recognizing the strong role of families and local communities in health-related issues.

To examine the trustworthiness of the study, Guba and Lincoln’s validation criteria were applied detailing the credibility, transferability, confirmability and dependability of the qualitative study design used. [28, 29]. Findings from this study, which are based on accurate analysis of the opinions of the study participants, form the basis of credibility. Method triangulation was applied when CB and KS coded and analyzed the data independently. Additionally, persistent observation took place involving CB, KS and RM researchers constantly reading and rereading the data, analyzing, theorizing and revising the concepts accordingly.

Regarding transferability, the data was studied until a conceptual idea emerged – in this case, that health literacy in Samoa is strongly influenced by the culture. By following the consolidated criteria for reporting qualitative research, thick descriptions of the themes and categories as well as a rich account of the participants and the research process were developed and outlined to enable others to assess whether the findings are representative of results in their own setting [30]. A gender bias should be noted as the majority of participants were women. The researchers took into account the complexity of the local setting and therefore followed a recruitment procedure in the villages through the local women’s communities. Secondly, the focus groups were conducted during the daytime, which is the time that most men are working in the fields to ensure food can be provided for their families. The villages from the sample are all in rural settings, but it should be noted that most of the country is rural as Samoa has only one city, which mainly serves as an administrative hub and accommodates the upper/middle class.

Dependability includes the aspect of consistency according to research standards. Furthermore, the study design was grounded in state-of-the-art knowledge of health literacy, with a specific conceptual focus derived from a validated European health literacy model. Finally, the principal investigator (CB) lived in Samoa at the time of the research and had obtained in-depth insights into local customs and traditions which were taken into account in the design and execution of the study. The third and fourth authors (RM, NdV) applied inquiry audit by reviewing the research process and data analysis made by the two initial coders (CB and KS).

Conformability refers to inter-subjectivity: interpretation of the data should be based on the data, not on the personal preferences of the researchers involved. An audit trail of field notes, logbooks and analytical notes was kept throughout the research process to facilitate transparency. With regards to reflexivity, the role of researchers and non-participants can be mentioned. The presence of non-participants (Ministry of Health) may have influenced the discussion, although the trained facilitator briefed the non-participants on their observer status. Nevertheless, caution was taken to ensure an open and transparent dialogue during the focus groups based on the semi-structured interview guide.

The inductive approach and the analysis applied, yielded the researchers to the emerging themes and categories. During the focus groups, themes became re-appearing and saturation was achieved at the analytical level. Probably more categories would have appeared with an increased number of focus groups, however, with the data available the seven presented categories emerged as a result of the thorough data analysis.

## Conclusion

Our study has shown that health literacy of Samoan is strongly influenced by cultural determinants, and there are seven broad categories that needs to be taken into account by policy-makers and public health experts when planning and conducting NCD interventions:: (1) Lack of personal ownership, (2) Family circle concerning health, (3) Role of community, (4) Basic noncommunicable diseases knowledge, (5) Medication adherence, (6) Traditional healing and (7) Perception of health system’s performance.

Increasingly, modern society puts an emphasis on the individual and person-centred health. Samoan culture puts an emphasis on family and interdependence between individuals and the community. These cultural concepts are essential for understanding health within the Samoan context and should be the baseline for public health practitioners to build their intervention upon. Health promotion materials or campaigns addressing the “individual” might not reach the intended audience as the individual does not see it as their responsibility or their personal commitment to act.

In the last ten years, high-level policy makers have been instrumental in driving forward the NCD agenda; however, the effects of these efforts have been limited, especially in the areas of NCD management and care. This study with novel evidence on health literacy in Samoa with regards to NCDs indicates that an understanding of the broader context should be integral in NCD management as health literacy, cultural adaption of public health interventions and health system performance play a central role. Future research could embark on this work and select NCDs cases in different stages of management, to learn and understand how health literacy could be an enabling factor in addressing and controlling this epidemic.

To make impact with public health interventions, it is crucial to understand, adjust and contextualize interventions aligned to the “health literacy” level of the targeted population.

The findings of this study provide evidence informed guidance how health literacy is influenced by culture and suggest that experts and policy makers employ participatory, culture-sensitive, public health interventions to address major public health problems as NCDs. The development of health literate, culture-appropriate, evidence-based public health interventions will be instrumental in reversing the NCD crisis in Samoa.

## Data Availability

The data from the focus groups interviews is available by contacting the corresponding author of this study, CB (c.bollars@maastrichtuniversity.nl). Additional data is being analysed as part of the public health interventions on NCDs for Samoa.

## Abbreviations

HLS-EU: European Health Literacy Project
NCDs: Noncommunicable diseases
WHO: World Health Organization
